# High school students’ mental health in the northern West Bank governorates in Palestine: A comparative study

**DOI:** 10.1097/MD.0000000000048393

**Published:** 2026-04-17

**Authors:** Mamoun S. Shawahna, Ahmad Hanani

**Affiliations:** a Psychology Department, Lincoln University College, Petaling Jaya, Selangor, Malaysia; b Public Health Department, Faculty of Medicine and Health Sciences, An-Najah National University, Nablus, Palestine.

**Keywords:** anxiety, cognitive well-being, depression, emotional equilibrium, psychological symptoms

## Abstract

Mental health issues have increasingly attracted scholarly attention due to their complex effects on individuals’ emotional, cognitive, and social functioning. In Palestine, prolonged conflict and exposure to instability have placed adolescents at elevated risk of psychological distress. This study aims to identify the mental health levels of high school students in the northern West Bank governorates of Palestine and to examine the impact of variables, including gender and field of study. A cross-sectional descriptive-analytical design was employed. Data was conveniently collected using the Brief Symptom Inventory-18. Out of 900 distributed questionnaires, 805 valid responses were analyzed using descriptive statistics and independent-samples *t* tests. Findings revealed moderate psychological symptoms among students, particularly in depression, somatic symptoms, and anxiety. Significant differences were found based on gender, with females reporting higher levels of psychological distress. Students on the literary track reported higher psychological symptom scores than those on the scientific track. Mean symptom scores were higher among literary-track students than scientific-track students (somatic: 1.27 vs 1.08; depression: 1.36 vs 1.16; anxiety: 1.26 vs 1.08; total: 1.29 vs 1.11). The study highlights the need to strengthen school-based mental health services in Palestine that align with students’ developmental stages and academic pressures, promoting early detection and prevention of psychological disorders.

## 1. Introduction

Palestine is an Arab Muslim country located in the Middle East, comprising 2 main districts: the West Bank and the Gaza Strip. The West Bank borders Jordan to the east, while the Gaza Strip borders Egypt to the south. For decades, the region has been under Israeli occupation, which has been accompanied by restrictions and chronic conditions.^[1]^

This prolonged reality has exposed Palestinians to cumulative social and psychological pressures. Continuous exposure to violence, insecurity, and political instability increases vulnerability to psychological disorders such as anxiety, stress, and depression, particularly among adolescents.^[2]^ Adolescence is a sensitive developmental stage during which emotional regulation and coping patterns are shaped, and susceptibility to environmental stressors is heightened, factors that may negatively influence academic performance, social adjustment, and overall mental well-being.

Mental health has therefore become a central topic of research due to the growing prevalence of psychological problems among youth.^[3]^ Rapid scientific, economic, and technological changes may further contribute to stress and poorer emotional adaptation.^[4]^ Limited mental health awareness can exacerbate the burden of disorders such as depression, anxiety, and stress.^[5]^ Emotional difficulties during adolescence have also been linked to behavioral and attentional problems such as hyperactivity.^[6]^ Importantly, psychological vulnerability in trauma-exposed groups may not be reflected only in diagnosable symptoms; It can also involve deeper disturbances in emotional awareness, including difficulties recognizing, labeling, and describing feelings (alexithymia). Such difficulties may reduce help-seeking and complicate emotion regulation. Clinical evidence indicates that greater trauma-related disorder severity and the alexithymia component “difficulty identifying feelings” are associated with higher levels of suicide ideation, suggesting a clinically meaningful pathway between trauma burden, impaired emotional awareness, and suicidal thinking.^[7]^

In addition, recent global crises, particularly the COVID-19 pandemic, have intensified psychological distress across populations, including adolescents. The pandemic has been associated with increased depressive symptoms and suicidal behavior, partly through social disconnection, disruption of daily routines, and declines in well-being and quality of life, with a disproportionate impact on vulnerable subgroups.^[8]^ Among secondary school students, these effects may be especially salient given the developmental sensitivity of adolescence to social isolation, academic pressures, and family stress, alongside limited access to mental health support in some settings.^[9]^

Adolescence is also a critical period during which many mental disorders first emerge.^[10]^ Students experiencing anxiety, depressive symptoms, or suicidal thoughts may be more likely to engage in high-risk behaviors, including smoking and substance use.^[11]^ Globally, approximately 14% of individuals aged 10 to 19 years’ experience some form of mental disorder.^[12]^ Social support plays a crucial role in mitigating these challenges and enhancing emotional well-being and resilience.^[13,14]^ Nevertheless, many students still lack access to adequate mental health services.^[15]^ Early intervention and awareness programs are therefore essential for adolescents’ coping skills. Social media platforms have become an important avenue for promoting mental health awareness,^[16]^ particularly among young people who prefer online counseling to overcome stigma or social anxiety.^[17]^ In Palestine, limited access to mental health services and ongoing political conflict further exacerbate stress, depression, and emotional distress among students.^[18]^ Related evidence on risky behaviors among Palestinian youth, especially in vulnerable environments such as refugee camps, also underscores the interplay between psychosocial distress and harmful behavioral patterns (e.g., high-risk substance use), reinforcing the need to prioritize adolescent mental health in school and community settings.^[9]^

Gender has also been recognized as an important factor influencing mental health outcomes. Differences in coping strategies and sociocultural expectations contribute to variations in emotional resilience and stress management.^[19]^ Female students tend to report higher levels of anxiety and depression,^[20]^ whereas males may demonstrate greater emotional regulation in some contexts.^[21]^ These patterns reflect a complex interaction of developmental, cultural, and contextual influences on adolescent mental health.^[22,23]^

Several theoretical frameworks have been proposed to promote adolescent mental well-being. Cavioni, Grazzani, and Ornaghi emphasized social-emotional learning and resilience as key approaches to addressing behavioral and emotional challenges.^[24]^ Participation in social-emotional learning programs enhances students’ emotional regulation, academic achievement, and interpersonal competence.^[25]^ Resilience-based interventions, such as yoga and cognitive-behavioral therapy, have also proven effective in reducing stress and anxiety among both teachers and students.^[26,27]^ Gender-responsive models, such as Ouriaghli Capability, Opportunity, and Motivation for Behavior behavioral change framework, aim to improve help-seeking behaviors among male students,^[28]^ whereas holistic approaches, such as Zhao multidimensional model, integrate the emotional, social, and spiritual domains of mental health.^[29]^ Classic frameworks by Caplan et al^[27]^ and Mrazek and Haggerty^[28]^ provided the foundation for modern preventive and rehabilitative practices.

Secondary school students in Palestine constitute a large segment of the educational population, underscoring the importance of ensuring that schools provide safe, supportive, and psychologically healthy environments.^[30]^ The secondary stage corresponds to adolescence, a period marked by physical, mental, and social maturation. Given the prolonged exposure to conflict, instability, and limited access to mental health services, investigating students’ mental health during this stage is essential.^[30,31]^

Accordingly, this study aimed to assess the overall mental health of secondary school students in the Northwest Bank governorates of Palestine and to examine differences by gender and academic track (scientific vs literary). Understanding these variations provides valuable insights for educators, policymakers, and healthcare professionals seeking to strengthen school-based mental health programs that align with students’ developmental and academic needs.

## 2. Methodology

This study is a cross-sectional descriptive-analytical design. This approach was considered most suitable for answering the main and sub-research questions and achieving the research objectives by validating the hypotheses through statistical measurements.

### 2.1. Sample population and sample size

The target population comprised Palestinian secondary school students enrolled in the 2023/2024 academic year in the northern West Bank governorates. A convenience sampling approach was used, and participants were categorized by sex and academic track (scientific vs literary). The minimum required sample size was estimated using the Raosoft online sample size calculator based on the total population of secondary school students in the study area. Assuming a 95% confidence level and a 5% margin of error, the minimum recommended sample size was 385. To ensure adequate statistical power for subgroup comparisons by sex and academic track and to account for potential nonresponse, 900 questionnaires were distributed. A total of 805 complete and valid questionnaires were returned and included in the final analysis (response rate 89.4%).

### 2.2. Study tools, validity, and reliability

The study employed the Brief Symptom Inventory (BSI-18), a standardized 18-item measure designed to assess psychological distress across 3 dimensions: somatic symptoms, anxiety, and depression, with 6 items representing each dimension. An Arabic version of the scale was used following translation and validation to ensure cultural appropriateness within the Palestinian context.^[32]^ Mean scores were interpreted using predefined cutoffs, whereby values of 0 to 1.33 indicated a low level, 1.34 to 2.66 a moderate level, and 2.67 to 4.00 a high level of symptom severity. The reliability of the BSI-18 was examined using Cronbach alpha coefficients. The results demonstrated acceptable to good reliability across the subscales (α = 0.799 for somatic symptoms, α = 0.799 for depression, and α = 0.811 for anxiety), and excellent reliability for the overall mental health scale (α = 0.930). These findings indicate strong internal consistency and support the instrument’s suitability for use in the current research setting.

### 2.3. Study procedure

Data was collected during the 2023/2024 academic year in secondary schools across the northern West Bank governorates. Prior to data collection, official approval was obtained from the administrations of the participating schools. Students were informed about the study objectives and procedures, and participation was voluntary. Informed consent was obtained from all participating students before completing the questionnaire, and anonymity and confidentiality were strictly ensured; no identifying information was collected, and responses were used for research purposes only and were not shared with school staff or other parties. Questionnaires were administered under standardized instructions, and returned forms were checked for completeness. Incomplete questionnaires were excluded, and valid questionnaires were coded and entered for analysis.

### 2.4. Bias

To reduce potential selection bias, recruitment aimed to include students from both the scientific and literary tracks and from both genders across the selected governorates. To minimize measurement bias, psychological symptoms were assessed using the BSI-18, a validated measure.

### 2.5. Statistical analysis

Data was analyzed using SPSS version 23 (IBM Corp., Armonk). Descriptive statistics were used to summarize participant characteristics and BSI-18 scores. Independent-samples *t* tests were performed to examine differences in mental health scores by sex and academic track (scientific vs literary). Homogeneity of variances was assessed using Levene test; when the assumption was violated, the Welch *t* test was applied. Statistical significance was set at *P* < .05.

## 3. Results and discussion

To address the first research question, descriptive statistics (means and standard deviations) were calculated for students’ responses across the study dimensions (Table 1). Overall, the findings indicate a moderate burden of psychological symptoms among secondary school students in the northern West Bank governorates. The total mean score (M = 1.55, SD = 0.85) suggests that psychological distress is present at a level warranting attention in the school setting, particularly during adolescence, a developmental period characterized by heightened vulnerability to emotional and behavioral difficulties and increased sensitivity to environmental stressors.^[33]^ Across dimensions, somatic symptoms recorded the highest mean (*M* = 1.81, SD = 1.22), followed by depression (*M* = 1.42, SD = 0.89), and anxiety (*M* = 1.42, SD = 0.78). The prominence of somatic complaints may reflect a common pattern in adolescent mental health whereby psychological distress is expressed through physical symptoms, especially in contexts where emotional disclosure is constrained by stigma or limited mental health literacy. This interpretation is consistent with evidence indicating that youth exposed to chronic adversity and conflict-related stress may exhibit elevated emotional distress alongside physical manifestations, and that depression frequently co-occurs with trauma-related symptomatology in conflict-affected settings.^[32]^ In this regard, the school context becomes central not only for academic development but also as a practical platform for early identification, supportive interventions, and referral pathways for students experiencing distress.^[33]^ While some international studies emphasize the role of the educational environment in promoting students’ psychological stability,^[29]^ caution is warranted when making direct comparisons across countries due to differences in sociocultural conditions, educational systems, and measurement approaches. Conversely, studies that highlight limited awareness and insufficient institutional support as barriers to student mental well-being align conceptually with the need to strengthen mental health promotion and support services within educational settings.^[34]^ Therefore, the current findings should be interpreted within the local context, including sociopolitical stressors and the availability of school-based support resources. Regarding item-level patterns (Tables 2 and 3), variability in endorsement suggests that students do not experience symptoms uniformly. Rather than interpreting individual items in isolation, it is more informative to view these patterns as indicating heterogeneity of symptom expression, where certain somatic or depressive experiences may be more salient for adolescents under chronic stress. This supports the adoption of multicomponent school-based strategies that address emotional regulation skills, stress management, and accessible counseling services.^[33]^ With respect to sex differences (Table 4), female students reported significantly higher mean scores than male students across all dimensions, indicating greater levels of somatic symptoms, depression, and anxiety. This pattern is consistent with broader adolescent mental health evidence showing that girls, particularly during mid-to-late adolescence, often report higher internalizing symptoms (e.g., anxiety and depression) than boys.^[33]^ Importantly, such differences should not be framed as inherent “emotional sensitivity” but rather as potentially reflecting a combination of developmental factors, gendered socialization, differential exposure to stressors, and variations in help-seeking or symptom reporting. The current results correspond with Campbell et al,^[21]^ who observed poorer mental health among adolescent girls than boys. However, the contrast with Haavik et al^[10]^ suggests that gender differences may vary across contexts and may depend on the constructs assessed (e.g., symptom reporting vs self-awareness and coping), sociocultural norms, and the availability of supportive environments. In Palestine, perceived stigma and barriers to mental health service utilization can further shape how students recognize and communicate distress; this is consistent with evidence documenting attitudinal and contextual barriers to mental health treatment among Palestinian students.^[30]^ Accordingly, schools should ensure that support systems are gender-responsive and accessible, while normalizing help-seeking and strengthening mental health literacy. Differences across academic tracks (Table 5) showed that students in the literary track reported significantly higher levels of depression, anxiety, and somatic symptoms than those in the scientific track. This finding suggests that academic specialization may be associated with differential stress profiles and psychosocial experiences. Crucially, the interpretation should remain consistent with the direction of the results: the literary track demonstrated higher symptom levels, and therefore, the explanation should consider factors that could increase distress in this group. Plausible hypotheses include differences in perceived academic and career opportunities, uncertainty regarding future pathways, variations in academic self-efficacy, and differential access to academic support. In some contexts, students’ perceived future prospects and social valuation of academic tracks can meaningfully influence emotional well-being during adolescence.^[33]^ These interpretations may also be shaped by local educational distributions and contextual pressures within the Palestinian schooling system.^[31]^ The discrepancy with Naceanceno et al,^[35]^ who reported no significant differences in anxiety across fields of study, may reflect differences in age group (college vs secondary students), educational context, or measurement, emphasizing that specialization-related effects are not universal and likely depend on contextual moderators such as coping strategies, social support, and institutional resources. Overall, the current discussion indicates that sex and academic track are significant correlates of psychological symptom burden among Palestinian secondary school students. These findings highlight the need for strengthening school-based mental health promotion and targeted support, including early screening, counseling services, psychoeducation, and referral pathways, especially in settings where stigma and barriers to treatment may limit access to formal mental health care.^[30]^ Given the known vulnerability of adolescents to mental health difficulties and the potential role of chronic stressors in shaping symptom expression, improving supportive school environments represents a practical and evidence-aligned strategy to mitigate psychological distress and promote resilience.^[33,32]^

**Table 1:** Mean scores and standard deviations for the sample responses on mental health level among secondary school students in Palestine.

Dimension	Mean score	Standard deviation	Percentage	Response level
1. Somatic Symptoms	1.8111	1.22409	60.3%	Medium
2. Depression	1.4178	0.89529	47.26%	Medium
3. Anxiety	1.4167	0.77593	47.22%	Medium
Overall score	1.5485	0.84975	51.6%	Medium

**Table 2:** Mean scores and standard deviations for responses on the somatic symptoms dimension, ordered in descending order.

Item		Mean score	Standard deviation	Percentage	Response level
10	A feeling of difficulty in breathing	3.57	5.844	89.2%	High
7	Nausea or abdominal cramps	1.75	1.409	43.7%	Medium
13	Nervousness, internal tremors, and tension	1.64	1.448	41.0%	Medium
4	Sensation of pain in the heart or chest	1.59	1.301	39.7%	Medium
1	Feeling of exhaustion, faintness, or dizziness, such as severe fatigue	1.55	1.207	38.7%	Medium
15	Episodes of fear and panic without reason	0.77	1.114	19.2%	Low
Overall score		1.8111	1.22409	45.2%	Medium

**Table 3:** Mean scores and standard deviations for responses on the depression dimension.

Item		Mean score	Standard deviation	Percentage	Response level
8	Feeling of rapid annoyance and agitation	1.76	1.59	44.0%	Medium
5	Nervousness, internal tremors	1.70	1.25	44.8%	Medium
14	Sudden fear or panic without reason	1.40	1.46	36.6%	Low
2	Feeling of fear	1.20	1.26	32.3%	Low
11	Episodes of fear and panic without reason	1.18	1.43	29.5%	Low
17	Feeling of instability to the extent that you cannot sit quietly in one place (you move excessively)	1.00	1.27	25.3%	Low
Overall score		1.41	0.89	35.4%	Medium

**Table 4:** Results of the independent samples *t* test for gender differences.

Dimension	Gender	Number	Mean	Standard deviation	*t*	Significance level
Physical symptoms	Male	442	1.0468	0.84161	‐4.726	0.000
	Female	363	1.3292	0.84562		
Depression	Male	442	1.1331	0.89914	‐4.448	0.000
	Female	363	1.4164	0.89935		
Anxiety	Male	442	0.9879	0.79823	‐6.933	0.000
	Female	363	1.4008	0.87420		
Total score	Male	442	1.0559	0.79442	‐5.689	0.000
	Female	363	1.3822	0.82179		

**Table 5:** Independent samples *t* test for field of study differences.

Dimension	Field of study	Number	Mean	Standard deviation	*t*	Significance level
Physical symptoms	Scientific	390	1.0756	0.85514	‐3.186	0.001
	Literary	415	1.2667	0.84459		
Depression	Scientific	390	1.1590	0.93868	‐3.090	0.002
	Literary	415	1.3566	0.87189		
Anxiety	Scientific	390	1.0821	0.88585	‐2.959	0.003
	Literary	415	1.2606	0.82231		
Total score	Scientific	390	1.1056	0.84625	‐3.272	0.001
	Literary	415	1.2946	0.78978		

## 4. Conclusion

The ongoing conflict and instability in Palestine continue to affect students’ psychological well-being. This study assessed the mental health levels of secondary school students in the northern governorates of the West Bank and found that the overall level of psychological distress was moderate. Significant differences were identified by gender, with females exhibiting higher levels of anxiety, depression, and somatic symptoms than males. Furthermore, differences were observed by academic track, with students in the scientific field reporting better mental health than their peers in the literary track. These results suggest that mental health among adolescents varies according to gender, academic environment, and contextual circumstances.

The study underscores the importance of school-based interventions and mental health promotion programs that enhance resilience, emotional regulation, and coping skills among Palestinian adolescents. Strengthening psychosocial support systems in schools is essential to prevent the escalation of mild distress into more severe mental disorders.

### 4.1. Recommendations

The study highlights the importance of strengthening school-based mental health services in Palestine. Programs should be designed to match students’ developmental stages and focus on building resilience and coping skills to manage anxiety, depression, and somatic symptoms. Increasing the number and effectiveness of trained school counselors is crucial for providing academic and emotional support. Moreover, schools should implement and evaluate preventive interventions to enhance students’ mental well-being. Special attention must be given to students on the literary track, who showed higher distress levels, through targeted counseling and support services. Ultimately, incorporating career guidance programs may help alleviate stress associated with academic specialization and enhance students’ psychological well-being.

### 4.2. Limitations

This study was geographically limited to the northern governorates of the West Bank, which may restrict the generalizability of its findings to other regions of Palestine. Data collection took place between December 20, 2023, and February 1, 2024, during the first semester of the 2023 to 2024 academic year, thereby establishing a specific temporal context. The research also focused on secondary school students in the Nablus governorate and thus did not include other educational levels or younger age groups. Conceptually, the study concentrated on 3 primary dimensions of mental health (somatic symptoms, anxiety, and depression) without exploring broader psychosocial or environmental variables. Furthermore, while gender and academic track were analyzed as primary comparison variables, other potentially influential factors, such as socioeconomic status, type of school, and parental education, were not examined due to ethical and logistical constraints in the school-based context. These unmeasured factors may have affected the interpretation of findings. However, schools from multiple governorates helped ensure a representative sample from diverse socioeconomic backgrounds, thereby enhancing the study’s external validity.

## Acknowledgments

The authors acknowledge Lincoln University College, Malaysia, and An-Najah National University in Palestine (www.najah.edu) for the technical support provided to publish this manuscript.

## Author contributions

**Conceptualization:** Mamoun S. Shawahna, Ahmad Hanani.

**Formal analysis:** Mamoun S. Shawahna.

**Investigation:** Ahmad Hanani.

**Methodology:** Mamoun S. Shawahna, Ahmad Hanani.

**Project administration:** Ahmad Hanani.

**Supervision:** Ahmad Hanani.

**Validation:** Ahmad Hanani.

**Writing – original draft:** Mamoun S. Shawahna, Ahmad Hanani.

**Writing – review & editing:** Ahmad Hanani.
